# Novel germline mutations in *FLCN* gene identified in two Chinese patients with Birt–Hogg–Dubé syndrome

**DOI:** 10.1186/s40880-016-0172-5

**Published:** 2017-01-09

**Authors:** Teng Li, Xianghui Ning, Qun He, Kan Gong

**Affiliations:** Department of Urology, Institute of Urology, National Urological Cancer Center, Peking University First Hospital, Peking University, No. 8, Xishiku Street, Xicheng District, Beijing, 100034 P. R. China

**Keywords:** Birt–Hogg–Dubé syndrome, The folliculin (*FLCN*) gene, Mutation, Renal cell carcinoma

## Abstract

Birt–Hogg–Dubé (BHD) syndrome, a hereditary renal cancer syndrome caused by mutations in the folliculin (*FLCN*) gene, is characterized by the presence of fibrofolliculomas, pulmonary cysts, spontaneous pneumothorax, and renal cell carcinoma (RCC). Few BHD syndrome cases have been reported in Asian countries, and cutaneous presentations are relatively rare in Asian patients. Asian BHD patients may be misdiagnosed due to their atypical manifestations. Here, we report two Chinese BHD patients with novel *FLCN* mutations (c.946-947delAG in exon 9 and c.770-772delCCT in exon 7). Both of them had RCC and spontaneous pneumothorax without fibrofolliculomas. In patients with RCC and pulmonary cysts but without cutaneous lesions, screening for mutations in the *FLCN* gene should be performed, especially for those with a family history of RCC or pulmonary cysts (pneumothorax).

## Background

Birt–Hogg–Dubé syndrome (BHD, OMIM #135150) is a rare autosomal dominant hereditary disease first described by Birt et al. in 1977 [1]. This syndrome is characterized by the presence of fibrofolliculomas (FFs), pulmonary cysts, spontaneous pneumothorax, and renal cell carcinoma (RCC) [2]. Germline mutations in the folliculin (*FLCN*) gene mapped to chromosome 17p11.2 were identified in BHD patients in 2002 [3]. *FLCN* mutations have also been detected in isolated familial spontaneous pneumothorax and familial clear cell carcinoma [4]. *FLCN* is considered a tumor suppressor gene and interacts with the mammalian target of rapamycin (mTOR) and adenosine monophosphate-activated protein kinase (AMPK) signaling pathways. The spectrum of *FLCN* gene mutations has been outlined in several reports and has been summarized in a database (http://www.lovd.nl/flcn) [5]; however, the genotype-phenotype associations between the *FLCN* gene and BHD syndrome are not well known.

Most BHD cases have been identified in Caucasians. Only several reports of compiled BHD cases in the literature have been from Asia, and most of them have been from Japan [6–8]. Many of the BHD patients from Asia have not had all symptoms (FFs, pulmonary cysts, spontaneous pneumothorax, and RCC) [8]. The most life-threatening manifestation of BHD syndrome is renal cancer, which is present in 27%–41% of patients [2, 9, 10]. RCCs in BHD patients can be multiple or bilateral and include various histopathologic types, such as hybrid oncocytic tumor, chromophobe RCC, clear-cell RCC, oncocytoma, and papillary RCC [10, 11].

Here, we report two Chinese BHD patients with two novel germline *FLCN* mutations. Analysis of these patients was approved by the Medical Ethics Committee of Peking University First Hospital. Written informed consent was obtained from the patients and their families.

## Case report

### Case 1

**Fig. 1:**
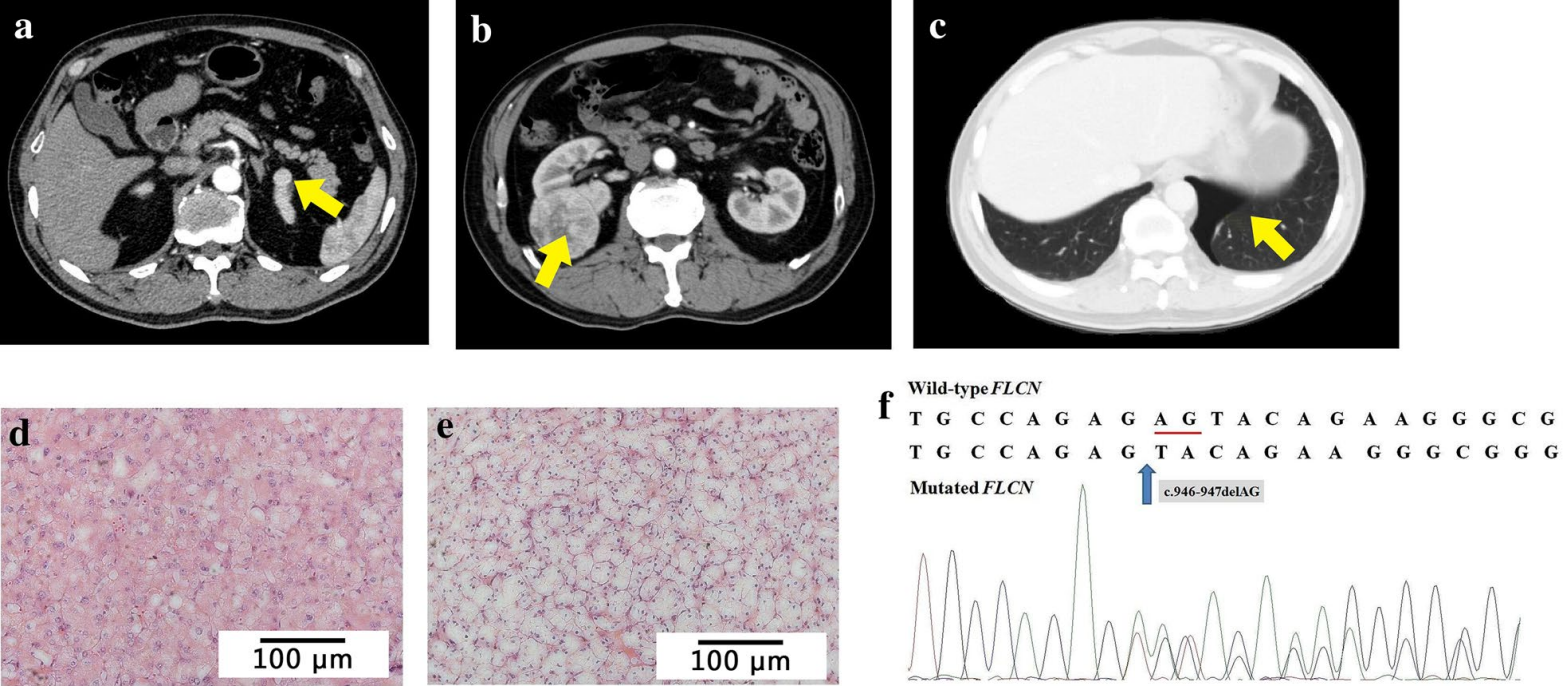
Lesions and folliculin (*FLCN*) mutation in case 1. **a** Abdominal computed tomography (CT) scan with intravenous contrast reveals a renal mass in the upper pole of the left kidney (*arrow*). **b** Abdominal CT scan with intravenous contrast reveals a renal mass in the right kidney (*arrow*). **c** CT scan shows left pneumothorax (*arrow*). **d** Histopathology of the left renal tumor shows a monotonous cellular pattern with mild nuclear pleomorphism and abundant, partly eosinophilic cytoplasm with a perinuclear halo (chromophobe renal cell carcinoma). **e** Histopathology of the right renal tumor shows clear cytoplasm surrounded by a distinct cell membrane (clear cell carcinoma). **f** Both the patient and his son have a deletion of AG (c.946-947delAG) in exon 9 of the *FLCN* gene, which causes a frameshift mutation starting at the 316th amino acid (p.316fs388x). The deleted bases are *underlined* in the normal sequence

A 54-year-old man was found to have asymptomatic, bilateral renal tumors by ultrasonography and computed tomography (CT) (Fig. 1a, b). CT scan also showed left pneumothorax (Fig. 1c). No apparent cutaneous lesions were found by careful inspection and palpation of the skin. The patient had a history of spontaneous pneumothorax at the age of 30. His son also had a history of spontaneous pneumothorax at the age of 17. Open bilateral and partial nephrectomies were performed. Histopathologic examination revealed that the tumor in the left kidney was chromophobe RCC, nuclear grade G2 (partial G1), and 1.7 cm × 1.5 cm × 1.5 cm in size (Fig. 1d); the tumor in the right kidney was clear-cell RCC, nuclear grade G2, and 5.0 cm × 4.7 cm × 4.5 cm in size (Fig. 1e).

Genomic DNA was extracted from blood samples collected from the patient and his son. Mutation examination revealed that the proband and his son carried a deletion of two bases (AG) at nucleotides c.946-947 in exon 9 (c.946-947delAG) of the *FLCN* gene (Fig. 1f), which caused a frameshift mutation starting at the 316th amino acid (p.316fs388x). No RCC was detected by abdominal CT scan on the son.

### Case 2

**Fig. 2:**
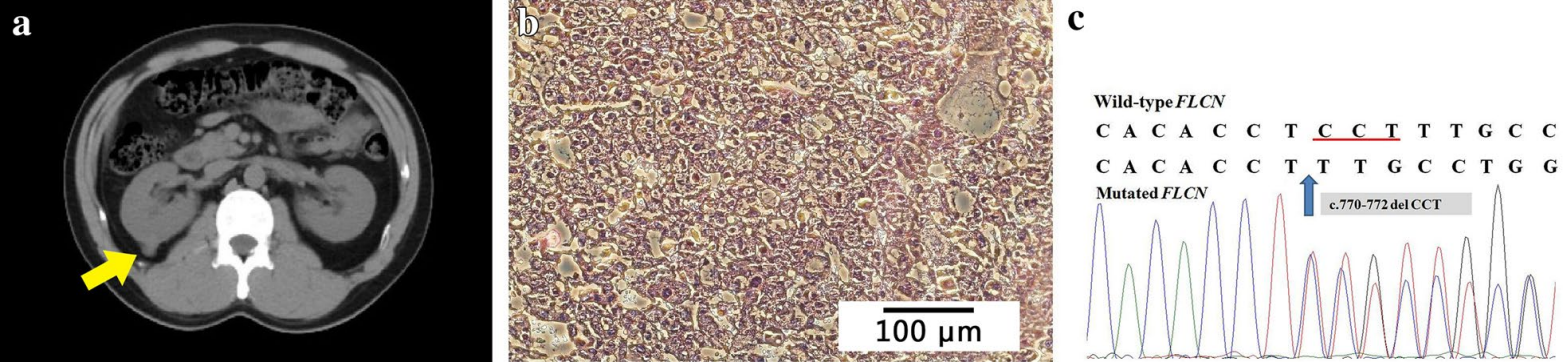
Lesion and *FLCN* mutation in case 2. **a** CT scan reveals a mass in the right kidney (*arrow*). **b** Histopathology of the resected tumor shows tumor cells with transparent, slightly reticulated cytoplasm with prominent cell membranes (chromophobe renal cell carcinoma). **c** The patient has a deletion of CCT (c.770-772delCCT) in exon 7 of the *FLCN* gene, which results in deletion of the 257th serine residue (p.257delS). The deleted bases are *underlined* in the normal sequence

A 37-year-old man presented with an asymptomatic right renal mass detected by ultrasonography and CT (Fig. 2a). No apparent cutaneous lesions were found by careful inspection and palpation of the skin. The patient had a history of spontaneous pneumothorax on both sides and underwent pulmonary bullectomy on the right side at the age of 30. His father had a history of recurrent spontaneous pneumothorax starting at the age of 28 and died of stoke at the age of 63. His uncle had a history of recurrent spontaneous pneumothorax starting at the age of 24 and died of an accident at the age of 56. Open partial nephrectomy was performed on the patient. Histopathologic examination revealed that the tumor was chromophobe RCC, nuclear grade G2, and 2.2 cm × 2.0 cm × 2.0 cm in size (Fig. 2b).

Genomic DNA was extracted from blood samples collected from the patient and his family members. Mutation examination revealed that the proband carried a deletion of three bases (CCT) at nucleotides c.770-772 in exon 7 (c.770-772delCCT) (Fig. 2c), resulting in deletion of the 257th serine residue (p.257delS). No *FLCN* mutations were found in any other members of the patient’s family.

## Discussion

Most reported cases of BHD have been from Western countries, and reports of cases from Asia are rare, likely due to the lack of awareness and atypical manifestations of this disease in Asian patients. Identification of BHD patients is usually based on dermatologic signs. However, Kunogi et al. [6] found that among 30 Japanese BHD patients, only 6 (20.0%) had cutaneous lesions, 1 (3.3%) was histologically diagnosed with FFs, and 29 (96.7%) had pneumothorax. Further, Furuya and Nakatani [7] reported that 13 (28.8%) patients had FFs, 40 (88.9%) had pulmonary cysts, and 9 (20.0%) had RCC among 45 BHD patients from 19 Japanese families. In addition, Murakami et al. [8] compiled 62 BHD cases from Asia and found that FFs were detected in 17 (27.4%), pulmonary cysts in 49 (79.0%), and RCC in 11 (17.7%). In comparison, Toro et al. [9] found that among 51 BHD families in the United States, 46 (90.2%) had FFs, 45 (88.2%) had pulmonary cysts, and 25 (49.0%) had renal tumors. Kluger et al. [12] reported 22 patients from ten unrelated families with BHD in France; 18 (81.8%) patients had FFs, 16 (72.7%) had pulmonary cysts or a history of pneumothorax, and 10 (45.5%) had renal tumors. Therefore, the incidence of FFs may be lower among Asian BHD patients compared with the higher incidence of 80%–90% reported among patients from the United States and Europe, whereas the pulmonary cyst incidences are similar between patients from Asian and Western countries. *FLCN* mutation screening is recommended for patients with RCC and pulmonary cysts without cutaneous lesions, especially for those with a clear family history of RCC or pulmonary cysts/pneumothorax.

In this report, case 1 had two pathologic types (chromophobe and clear-cell) of RCC. BHD patients are each likely to have various types of RCC [13], especially those with tumors with composite oncocytoma or chromophobe RCC histology [14]; these pathologies include the known familial hereditary RCCs, such as von Hippel-Lindau (VHL) syndrome, BHD syndrome, hereditary papillary renal cell carcinoma (HPRCC), hereditary leiomyomatosis renal cell carcinoma (HLRCC), and tuberous sclerosis complex (TSC). Pavlovich et al. [10] summarized clinical data of 124 BHD patients with 84 resected renal tumors, of which 56 (67%) were hybrid oncocytic tumors, 19 (23%) were chromophobe RCC, 6 (7%) were clear-cell RCC, and 3 (4%) were renal oncocytoma. Urologists should thus be aware that the presence of chromophobe/oncocytic renal neoplasms, especially those that are bilateral or multifocal, may indicate a diagnosis of BHD syndrome, even if dermatologic lesions are absent [11].

Over 100 unique mutations in the coding region of *FLCN* have been identified. Most of these mutations are frameshift, nonsense, insertion/deletion, or splice site mutations, resulting in truncation and inactivation of the encoded protein folliculin [15]. However, no clear genotype–phenotype associations between *FLCN* mutation type or location and skin, lung, or renal manifestations have been identified to date; only a significantly lower frequency of renal neoplasia has been reported in patients with a deleted cytosine in the exon 11 mutational “hot spot” compared with the patients with an inserted cytosine at this location [15, 16]. A definite diagnosis of BHD can only be made when a pathogenic germline *FLCN* mutation is detected. Detection of an *FLCN* gene mutation not only confirms the diagnosis in suspected patients but also enables the diagnosis of at-risk family members with this disease. Once RCC is detected in BHD patients, it can be closely monitored as long as the dominant lesion is less than 3 cm in diameter. Surgical resection is recommended when the solid lesion or solid portion of a mixed lesion exceeds the threshold diameter of 3 cm [10]. For patients with bilateral renal tumors measuring greater than 3 cm, staged surgeries in two separate settings are recommended [11]. The tumor growth rate and location are additional factors that should be considered [2, 11]. Recently, a kidney-specific knockout model was developed by disrupting mouse *FLCN* in proximal tubules [17]. This model provides a valuable tool for further study of *FLCN*-deficient renal tumorigenesis and for tests of new drugs (such as mTOR inhibitors) for the treatment of BHD-associated renal tumors.

Here, we report two Chinese patients with RCC and spontaneous pneumothorax who were diagnosed with BHD syndrome. Both patients underwent *FLCN* gene mutation screening and were found to have two novel mutations (c.946-947delAG and c.770-772delCCT) that have not been reported previously. Importantly, skin FFs were absent in both patients. These findings demonstrate, particularly to urologists, the importance of performing *FLCN* gene mutation screening for patients with RCC and pulmonary cysts.
